# Hansen’s Disease: A Complete Clinical Guide

**DOI:** 10.3201/eid3101.241121

**Published:** 2025-01

**Authors:** Rezhan Hussein

**Affiliations:** Pennsylvania State Milton Hershey Medical Center, Penn State College of Medicine, Hershey, Pennsylvania, USA

**Keywords:** Hansen’s disease, *Mycobacterium leprae*, *Mycobacterium leptomatosis*, bacteria, leprosy, mycobacteria

*Hansen’s Disease: A Complete Clinical Guide* is an excellent resource for one of the most disabling and neglected tropical diseases, Hansen’s disease (leprosy) (Figure). The book is an easy-to-read yet powerful resource for patient care, public health planning, and future directions for Hansen’s disease research.

**Figure:**
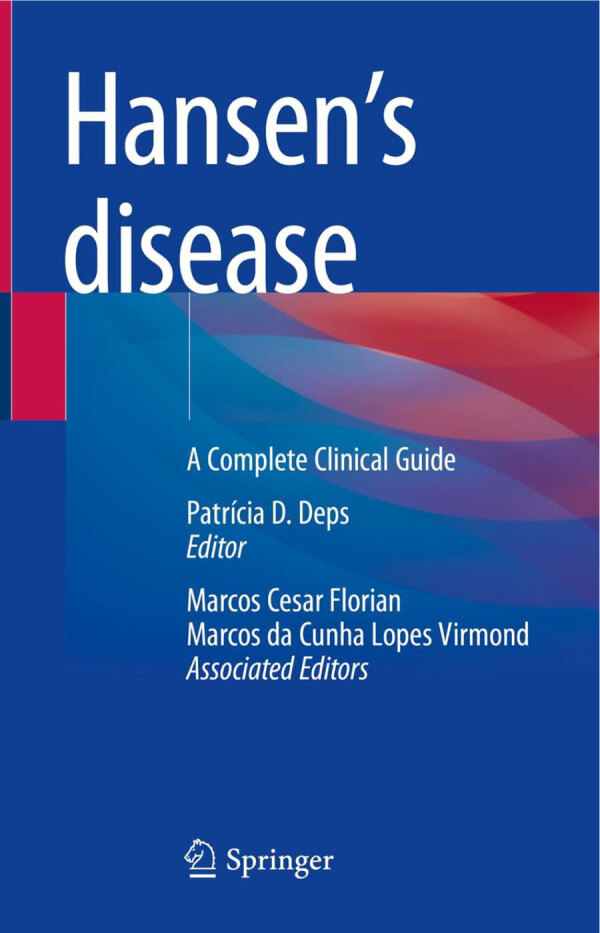
Hansen’s Disease: A Complete Clinical Guide

The journey of disease recognition, failures, frustrations, and breakthroughs in the management of Hansen’s disease are very well presented. The metabolic, genetic, and immunological mechanisms in susceptibility to leprosy are of particular interest. The new and advanced methods of detection and identification of *Mycobacterium leprae* and *Mycobacterium lepromatosis* are valuable. The book has a comprehensive differential diagnosis for skin lesions that might mimic leprosy lesions and includes high quality-colored images and figures.

The consequence of co-infection and immunosuppression with other tropical diseases and tuberculosis is described. Various ocular manifestations of Hansen’s disease that might not be recognized as typical, which includes chronic iridocyclitis that almost exclusively occurs in multibacillary disease, are highlighted, in addition to the more known cutaneous and neurologic manifestations of leprosy. The diagnostic section of the book includes high resolution ultrasound and radiologic images.

*Hansen’s Disease: A Complete Clinical Guide* also focuses on the complications and disabilities caused by Hansen’s disease and the associated stigma and psychosocial aspects experienced by patients. The book includes a story from the second author, who has had Hansen’s disease himself, to emphasize the role of healthcare providers in addressing those aspects of the disease.

Immunoprophylaxis with the bacillus Calmette-Guérin vaccine and chemoprophylaxis with rifampin are needs in endemic areas that might provide hope of transmission interruption. Those needs will also aid in reaching the World Health Organization’s goal of Hansen’s disease eradication.

Because the readers of *Hansen’s Disease: A Complete Clinical Guide* are mostly outside the United States, the book did not highlight valuable resources for clinicians in the United States. The National Hansen's Disease Program is the epicenter of Hansen’s disease care, research, and information in the United States. The program reviews tissue biopsies, provides free medications to patients, and offers guidance and support to clinicians.

An amazing and demanding amount of work was put into writing *Hansen’s Disease: A Complete Clinical Guide*. This book serves as a complete reference for Hansen’s disease and appeals to most healthcare providers, especially internists, dermatologists, ophthalmologists, neurologists, and radiologists because they are on the frontline of Hansen’s disease care and should consider leprosy when evaluating patients. Early diagnosis is necessary to minimize the risk of transmission and long-term disabilities.

